# Supplementary material for: Scube2 primes Dispatched and ADAM10-mediated Shh release by recruiting HDL acceptors to the plasma membrane
Source: Commun Biol. 2026 Jan 8;9:189. doi: 10.1038/s42003-025-09466-x (PMC12881369; doi:10.1038/s42003-025-09466-x)
Supplement: Supplementary file 3 — Description of Additional Supplementary files [file 42003_2025_9466_MOESM3_ESM.pdf]

Description of Additional Supplementary files

File name: Supplementary Data 1

Description: Source data behind the graphs in this paper

File name: Supplementary Data 2

Description: Complete list of Scube2 interacting serum proteins, as determined by mass spectrometry
