# Supplementary material for: Scube2 primes Dispatched and ADAM10-mediated Shh release by recruiting HDL acceptors to the plasma membrane
Source: Commun Biol. 2026 Jan 8;9:189. doi: 10.1038/s42003-025-09466-x (PMC12881369; doi:10.1038/s42003-025-09466-x)
Supplement: Supplementary file 6 — Reporting summary [file 42003_2025_9466_MOESM6_ESM.pdf]

nature portfolio

Corresponding author(s): Dr. Kay Grobe

Last updated by author(s): Nov 24, 2025

Reporting Summary

Nature Portfolio wishes to improve the reproducibility of the work that we publish. This form provides structure for consistency and transparency in reporting. For further information on Nature Portfolio policies, see our Editorial Policies and the Editorial Policy Checklist.

Statistics

For all statistical analyses, confirm that the following items are present in the figure legend, table legend, main text, or Methods section.

n/a	Confirmed	
☐	☒	The exact sample size (n) for each experimental group/condition, given as a discrete number and unit of measurement
☒	☐	A statement on whether measurements were taken from distinct samples or whether the same sample was measured repeatedly
☐	☒	The statistical test(s) used AND whether they are one- or two-sided<br>*Only common tests should be described solely by name; describe more complex techniques in the Methods section.*
☒	☐	A description of all covariates tested
☒	☐	A description of any assumptions or corrections, such as tests of normality and adjustment for multiple comparisons
☐	☒	A full description of the statistical parameters including central tendency (e.g. means) or other basic estimates (e.g. regression coefficient) AND variation (e.g. standard deviation) or associated estimates of uncertainty (e.g. confidence intervals)
☐	☒	For null hypothesis testing, the test statistic (e.g. F, t, r) with confidence intervals, effect sizes, degrees of freedom and P value noted<br>*Give P values as exact values whenever suitable.*
☒	☐	For Bayesian analysis, information on the choice of priors and Markov chain Monte Carlo settings
☒	☐	For hierarchical and complex designs, identification of the appropriate level for tests and full reporting of outcomes
☒	☐	Estimates of effect sizes (e.g. Cohen's d, Pearson's r), indicating how they were calculated

Our web collection on statistics for biologists contains articles on many of the points above.

Software and code

Policy information about availability of computer code

Data collection	Nikon F-package software for automatic counting of ommatidia V.4.5.01, MoticImage V.2.0, Leica Application Suite X V. 3.7.1.21655, Zeiss ZEN V.3.6 and QSoft401 V. 2.7.3.883, BD Accuri C6 flow cytometer (BD Biosciences)
Data analysis	Prism10, Version 10.6.1 (799), Fiji/ImageJ 2.16.0 1.54g, FlowJo single cell analysis software.

For manuscripts utilizing custom algorithms or software that are central to the research but not yet described in published literature, software must be made available to editors and reviewers. We strongly encourage code deposition in a community repository (e.g. GitHub). See the Nature Portfolio guidelines for submitting code & software for further information.

Data

Policy information about availability of data

All manuscripts must include a data availability statement. This statement should provide the following information, where applicable:

- Accession codes, unique identifiers, or web links for publicly available datasets
- A description of any restrictions on data availability
- For clinical datasets or third party data, please ensure that the statement adheres to our policy

Source data are provided with this paper. All requests for materials and correspondence should be addressed to K.G.

Research involving human participants, their data, or biological material

Policy information about studies with human participants or human data. See also policy information about sex, gender (identity/presentation), and sexual orientation and race, ethnicity and racism.

Reporting on sex and gender	n/a
Reporting on race, ethnicity, or other socially relevant groupings	n/a
Population characteristics	n/a
Recruitment	n/a
Ethics oversight	n/a

Note that full information on the approval of the study protocol must also be provided in the manuscript.

Field-specific reporting

Please select the one below that is the best fit for your research. If you are not sure, read the appropriate sections before making your selection.

☒ Life sciences ☐ Behavioural & social sciences ☐ Ecological, evolutionary & environmental sciences

For a reference copy of the document with all sections, see nature.com/documents/nr-reporting-summary-flat.pdf

Life sciences study design

All studies must disclose on these points even when the disclosure is negative.

Sample size	Sample-size estimation was not required, as statistical assessment (post-hoc tests) of generally closely comparable wing/eye phenotypes gave significant results using the (large) datasets used. We detected strong differences between the genotypes analyzed; post-hoc tests confirmed that sample sizes were sufficient for analysis.
Data exclusions	None
Replication	Replication was not required, due to the consistent phenotypes (wing/eye phenotypes) obtained in our study. Clonal analyses in Drosophila also gave highly consistent findings due to the "Minute" technology used in our study (allowing to study only the genotype of interest in a clean null background)
Randomization	no group allocation was performed in this study and randomization was therefore not required
Blinding	Yes, technical personnel doing the quantification of eye phenotypes were not informed about their genotypes nor any expected outcomes (specimen were numbered). Blinding was performed during collection and analysis.

Reporting for specific materials, systems and methods

We require information from authors about some types of materials, experimental systems and methods used in many studies. Here, indicate whether each material, system or method listed is relevant to your study. If you are not sure if a list item applies to your research, read the appropriate section before selecting a response.

Materials & experimental systems

n/a	Involved in the study	
☐	☒	Antibodies
☐	☒	Eukaryotic cell lines
☒	☐	Palaeontology and archaeology
☐	☒	Animals and other organisms
☒	☐	Clinical data
☒	☐	Dual use research of concern
☒	☐	Plants

Methods

n/a	Involved in the study	
☒	☐	ChIP-seq
☐	☒	Flow cytometry
☒	☐	MRI-based neuroimaging

Antibodies

Antibodies used	2A1 anti-Ci and anti-Shh 5E1 (Developmental Studies Hybridoma Bank), rabbit-anti-Shh (Cell signaling C9C5), rabbit-anti-GAPDH (Cell Signaling, GAPDH 14C10, #2118), anti-actin (Sigma-Aldrich, A3854), anti-FLAG antibodies (Sigma, St. Louis, USA), anti-ApoA1 and ApoE4 antibodies (ABIN7427912, antibodies-online.com).
Validation	All commercial antibodies were verified by the suppliers and gave specific signals in our study. DSHB antibodies also gave specific signals as shown in this study and have also been tested by the depositors (2A1: PMID: 7577671 and 10H6: PMID: 9056773).

Eukaryotic cell lines

Policy information about cell lines and Sex and Gender in Research

Cell line source(s)	Bosc23 cells (RRID: CVCL_4401) from D. Robbins, University of Miamy, USA. C3H10T1/2 cells were obtained from the University of Braunschweig, Germany. HEK293 cells were obtained from C. Garbers, University of Hannover, Germany.
Authentication	Bosc23 cells, a HEK293 derivative, were validated using PCR single locus technology (eurofins, sample # CL180215_018). C3H10T1/2 reporter cells were verified by Shh-induced differentiation into alkaline phosphatase-producing osteoblasts. HEK293 cells were not authenticated.
Mycoplasma contamination	All three cell lines tested negative for mycoplasma (PCR)
Commonly misidentified lines (See ICLAC register)	not found

Animals and other research organisms

Policy information about studies involving animals; ARRIVE guidelines recommended for reporting animal research, and Sex and Gender in Research

Laboratory animals	Drosophila melanogaster (individual fly genotypes as outlined in the Materials section)
Wild animals	no wild animals were used in the study
Reporting on sex	both sexes were considered in the study
Field-collected samples	no field collected samples were used in the study
Ethics oversight	no ethical approval was required for this study

Note that full information on the approval of the study protocol must also be provided in the manuscript.

Plants

Seed stocks	none were used in this study
Novel plant genotypes	n/a
Authentication	n/a

Flow Cytometry

Plots

Confirm that:

- [x] The axis labels state the marker and fluorochrome used (e.g. CD4-FITC).
- [x] The axis scales are clearly visible. Include numbers along axes only for bottom left plot of group (a 'group' is an analysis of identical markers).
- [x] All plots are contour plots with outliers or pseudocolor plots.
- [x] A numerical value for number of cells or percentage (with statistics) is provided.

Methodology

Sample preparation	were transfected with Scube2 and a Scube2 variant lacking the major HS-binding amino acid motif, non-enzymatically removed from the culture dish by using Versene (PAA), and suspended in PBS containing 5% FCS in a total volume of 0.5 ml. Scube2-transfected cells were incubated with heparinases I to III (AMS Biotechnology) at 37°C or with 10 µg/ml heparin (AppliChem) at 4°C for 1 h. Cells were washed and treated with -FLAG antibody (1:500 dilution) for 1 h and fluorescein isothiocyanate-conjugated goat- -rabbit secondary antibody (1:200 dilution, Dianova) for 30 min on ice.
Instrument	BD Accuri C6 flow cytometer (BD Biosciences)
Software	FlowJo single cell analysis software
Cell population abundance	Relative abundances of FITC signals are shown in the histogram plots (Supplementary Figure 7A), negative control cells lacking the ligand are included in all experiments to demonstrate specificity of the signals.
Gating strategy	Gating was performed for live cells.

☐ Tick this box to confirm that a figure exemplifying the gating strategy is provided in the Supplementary Information.
